# Family violence against children and adolescents in context: How the territories of care are imbricated in the picture**^1^**

**DOI:** 10.1590/1518-8345.0593.2735

**Published:** 2016-08-08

**Authors:** Diene Monique Carlos, Maria das Graças Carvalho Ferriani

**Affiliations:** 2 Post-doctoral fellow, Escola de Enfermagem de Ribeirão Preto, Universidade de São Paulo, PAHO/WHO Collaborating Centre for Nursing Research Development, Ribeirão Preto, SP, Brazil.; 3 PhD, Full Professor, Escola de Enfermagem de Ribeirão Preto, Universidade de São Paulo, PAHO/WHO Collaborating Centre for Nursing Research Development, Ribeirão Preto, SP, Brazil.

**Keywords:** Family, Child, Adolescent, Domestic Violence, Primary Health Care

## Abstract

**Objective:**

to understand the context of care addressed to the families involved in family violence against children and adolescents (IVCA), as produced in the context of the Primary Health Care (PHC), from the vantage point of the practitioners of a municipality in the State of Sao Paulo.

**Methods:**

qualitative research of the social-strategic type, based on the Complexity Paradigm. The participants were 41 health practitioners in five health units of the municipality under study, pertaining to the five districts of the municipality. Data collection was done through 5 focus groups and 10 semi-structured interviews from April 24th 2013 to December 12th 2013. Data analysis was oriented by the comprehension and contextualization mindset and based on the dialogic, recursive and hologramatic principles.

**Results:**

two main issues regarding the care provided by the Health of the Family team were identified: the context of this violence (the domestic space) and the power relations that prevail in the territory where this violence surfaces. The community health workers are the targets of specific attention because they experience the live/work dialogic in this same area.

**Conclusion:**

paying attention to the territory, and considering the complexity of contexts and dimensions is inherently linked to the design of care to families involved in IVCA in the PHC environment.

## Introduction

Violence is responsible for more than 1 million deaths per year, a 2,5% of total world mortality. Notwithstanding this fact, these numbers only represent the top of a pyramid that better represents the size of this issue. Thousands of individuals worldwide are non-fatal victims of violence every day, representing the bottom of this pyramid. Children and adolescents are a large part of this silent victims^1^. This phenomenon is still more complex due to the fact that its main scene - the family space - and the repeated occurrence, hampers the care of the practitioners that give services to these families^2^ ^-^ ^3^. 

The Ministry of Health has the proposal of integration of resources and services for caring for these families involved in family violence against children and adolescents (IVCA) within a network of care, in an inter-institutional and inter-sectorial rationale, guides by Primary Health Care (PHC)^2^ ^-^ ^3^. PHC is the first level of care and the preferred gateway of the Brazilian National Health System (SUS). It is understood that, due to geographic proximity to populations, it has the best conditions to identify, welcome and build therapeutic projects addressed to families involved in IVCA, along with the rest of the service network^2^.

Recent studies approaching the subject of violence experienced by children and adolescents, remark the importance of the contextual factors of these stakeholders in coping with violence, and cite as the main factors, the family, peers and community; showing the alienation of the structures of child and adolescence protection, using comprehensiveness as a working instrument ^4^ ^-^ ^7^. For this purpose and trying to overcome the challenges in the healthcare area, specifically for nursing care related to IVCA, we state our guiding interrogations: Understanding PHC as the axis of the network of care for IVCA, how is the context where this care is provided? Which are the facilitators and impeding factors for caring in the context of life of the families and work of the providers? 

From the standpoint of these interrogations, the object of this study was the context of caring for the families involved in IVCA, within PHC. The Complexity Paradigm, using Edgar Morin's concepts, one of its main authors, as a reference was used as foundation for comprehension of this object. Complex thinking is proposed as an approach for objects that are "weaved together", trying to consider distinct, sometimes contradictory parts, as imbricated in the full picture of a phenomenon, as inserted in a context and not in isolation, in a dialogic perspective. For this purpose, it is needed a multi-view look into this phenomenon, trying to perceive and link local, global and the multiple relations among the parts, the whole and the context, allowing for a multi-dimensional approach, that may be contextual, dynamic and trans-disciplinary, including the contradictions^8^. To look at IVCA in a multi-dimensional way and to comprehend care in the context where that care really happens, may guide the potentialities and the impediments that the PHC practitioners face in this situation. 

For these reasons, the objective of this study was to understand the context of care that is delivered to the families involved in IVCA, as produced in PHC, from the point of view of the professionals of the Health Basic Units (HBU) of a municipality in the State of Sao Paulo

## Methodology

Qualitative approach study, using strategic social research, with foundations in the Complexity Paradigm, as already stated. The methodological pathway was oriented by the mindset related to comprehension and conceptualization, present in the Complexity Paradigm. Contextualization means to recur to strategies that aim to understand a certain phenomenon as linked and not in isolation. Comprehension tries to apprehend the meaning of an object or event and its relations with other objects or events^9^.

Other than these notions, we used the principles of complexity, such as: dialogic, recursive and hologramatic. The first implies the joining and association of contradictory factors in the analysis of a certain phenomenon. The second principle is related to the recursive action in the organization, as in a whirlwind, remarking that "the recursive process is a process in which products and effects are at the same time causes and producers of the same factor that produced them"^8^. The third principle is represented by an hologram image, in which each point contains almost all the information on the represented object, in a way in which not only the part is in the whole, but the whole is inserted in some way, in the part^8^ ^-^ ^9^.

The field of study was a large municipality in the State of Sao Paulo. The complexity of the municipal health system lead to a division in health districts, in a progressive decentralization process of the planning and management of health, in areas of about 200.000 pop. In this fashion, the municipality is divided in five Health Districts, North, South, East, Northwest and Southwest.

The municipality under study has 62 HBU's organized through the Health of the Family Strategy (HFS). There is a HBU for approx. each 20.000 pop. with multiprofessional teams including physicians of the basic specialties (clinicians, pediatricians, ObGyn), nurses, dentists, nursing auxiliaries and dental assistants. A few HBU's also have mental health staff (psychiatry, psychology and occupational therapy). 

The participants in this study were the workers of the HBU's with the following inclusion criteria: (i) one unit from each district was included, according to availability and authorization from the district and local coordinator; (ii) only the workers with at least one year working in the Unit were considered and also they needed to be involved in actions related to families involved in IVCA; (iii) to include enough number of participants to find re- incidence and theoretical saturation of the information. The saturation criteria for the sample were guided by the answers to the objective of the research, and with a larger emphasis in deepening the guiding issues for comprehension and contextualization of the topic.

In this way and contingent to availability, local and district organization of the units, five HBU were selected and their practitioners were selected by the teams themselves, the number of practitioners and professions varying in each unit. The number and type selected was: 41 practitioners, from them 17 community health workers (CHW); 4 nursing auxiliaries; 4 nurses; 1 occupational therapist; 4 pediatricians; 2 psychologists; 1 psychiatrist; 2 clinicians; 1 dental health assistant; 5 multiprofessional health residents. The participants were 6 men and the remaining women, and mainly concentrated in the age group from 31-40 years old.

The contact with the health district of the municipality began in January 14^th^ 2013 with the objective of discussing and selecting the participant HBUs. After this definition, following availability and local team organization, the meetings for data collection were scheduled. The participants HBUs were designated by the numbers 1 through 5, following the order of the data collection, to keep their anonymity. Data collection went from April 24^th^ to December 17^th^ 2013. 

Data collection happened through focus groups and semi-structured interviews. The focus groups were guided by the following question: How is delivered care to families involved in IVCA? From this trigger, the debates were launched oriented to comprehension and contextualization of this topic. There were two meetings in each Unit, of approx. 90 minutes, in a room with good acoustic, lighted and free of interruptions. As stated in the literature, groups had a rapporteur, an observer and a moderator, and were identified as Gp1, Gp2, Gp3, Gp4 and Gp5, in the sequence that they were happening.

Later on, the interviews were conducted with the same participants in different days. The selection of providers was oriented by the heterogeneity of the different professional groups. The guide for the interview had three open, guiding questions to allow the interviewee to have a specific look on the object of study and to reduce the interference of the interviewer, in this case: When facing a suspect or confirmed case of IVCA, how does the team acts? Tell me about the main impediments to act in these situations? And what about the facilitators?

The interviews lasted for approx. 20 minutes and were coded with E and numbered following the sequence: E1, E2, E3 and so on. The profession of the practitioners was not noted, and was recorded only when there was specific information that could contribute for the diagnosis. Through a process analysis of the data, the researchers reached the saturation point after the 10^th^ interview, and at that moment no more informants were included in the study. The reports were recorded in the *Easy Voicer* software in a machine with MP5 technology, then transferred to computers and transcribed in full.

Data analysis was guided by the following steps^10^: classification and organization of the information through careful reading and identification of the main points made by the interviewees and group debates, referencing to the pertinence and relevance for the object under study; organization of referential frames with the main points of the answers of the practitioners to gather a full view of the information in order to categorize them; and establishing of relations among pieces of data through organization in categories, groups of elements, ideas or expressions around concepts that may encompass all these aspects. Subsequently, an effort was made to relate those data and the complexity paradigm (Specially the ideas of comprehension and contextualization and the dialogic, recursive and hologramatic principles), the legal provisions and the literature around care networks for families involved in IVCA. The emergence of the context in which this care is produced through PHC, the living territory of these families, was the category that guided the present study.

The study was submitted to be analyzed by the Committee for Research Ethics of the Nursing School of Ribeirao Preto of the University of Sao Paulo, following Resolution 196/96 proposed by the National Health Council of the Ministry of Health, approved on November 14^th^ 2012, by protocol CAAE 01726512.0.00005393, letter 217/2012. Also, following this resolution, a spontaneous consent was required through signing a Free and Informed Consent Form (FICF). The Municipal Health Secretariat of the municipality approved this study on May 18^th^ 2012. The district and local authorities of the Units also approved the study.

## Results and discussion 

The IVCA phenomenon is complex and interferes strongly in human relations. Looking at this complexity and at the context of actions and care for this phenomenon object of the study (PHC) it is possible to understand the imbrications of the territories (Of action, care, life, conviviality) and the relations among them, specifically power relations as intrinsic and inherent to this study. 

The Health Unit is a reference for health care of the population, as long as the community get ownership in it, feeling it as inherent to their living territory. This Unit surfaces frequently as the only space where families are heard: *Because I see this Health Unit not only as a Health Unit, it is a place where people of the community can come together to tell each other their problems, all kind of problems (...) Is our closest place* (Gp1). Literature on violence against children and adolescents, in the last three years shows that integrating different social services for this population and their families is still a challenge. The proposal of addressing the determinants and conditions of health that influence production and re-production of violence implies building an interdisciplinary and inter-sectorial look geared towards these factors. As it is seen in the group speech, the Health Unit is seen in an isolated fashion. Several studies stress that the initiatives that promote the development of children and adolescents should be integrated into strategies for violence prevention that involve the stakeholders under a comprehensiveness umbrella, and the international organizations should speed up the evidence-based knowledge diffusion, and fostering innovating practices in caring for IVCA^5^ ^-^ ^11^.

The CHW's merit special attention in the context of caring for IVCA. These practitioners are the links between the population and the health team, and live a dialogic^8^ relation being at the same time the ones that offer care directly to the population and also they are part of the community: *We have that side of us, being part of the population where we work, right?... There is this side* (E 5).

Legislation specifies the requisites for the CHW work, among them the possibility of living in the same area where he/she works. A study about care for pregnant teenagers under the perspective and action of rhe CHW's showed that because of them living in the same community, there was a trust and intimacy relationship between the CHW's and the teenagers, fostering closer link to the Health Unit^12^. It is understood that to live in the territory is important for getting a better grasp of the context, and more chances of planning actions with impact and improvements of the quality of life of the population. However, there are a few caveats, especially in coping with situations involving violence. Literature shows criticism to the criterion of the CHW mandatory living in the same area of work, connecting this fact with physical and psychical involved in of them. Not having permanent education activities for these practitioners, that do not have *a priori* higher diplomas, is not done or done through initial approaches^12^ ^-^ ^13^.

The topic so far analyzed should be expanded beyond the understanding of the territory as a geographic definition, of a political-administrative nature, that give boundaries to the action of a certain health service. In Social Sciences, the concept of territory assumes three suppositions. Firstly, there is a difference between geographic space and territory, Secondly; there is a historical construction of the territory, in the social field, linked to power relations. And thirdly, the more relevant for this study, is the subjective dimension of the territory, identification and domination of a certain space and in the objective dimension, related to political and economical action. Following this view, territories are defined by the power as effective in that space, not only political but also de symbolic appropriation^14^ ^-^ ^15^. 

The violence as phenomenon acts upon this territory and (trans)forms it through a reciprocal relationship. This study pays attention to the implication of the territory(ies) in caring for families involved in IVCA. Two key questions were identified for this care done through the family care team and specially by the CHW's: the context where this violence happens and the existent power relations in the mergence of this care in the territory.

The domestic context is presented as sacred, veiled by privacy. In this specific milieu, the contradictions and wider social consequences exist, deepening inequalities and mistakes, translated into lack of resources to cope with daily situations, stress, oppression and weak and unfavorable conditions for healthy family relations^3^. Going deeper in these relations was understood by some practitioners as an invasion: *There is a boundary (...) I cannot stop the mother's decision. We don't know how far can we go?(...)We cannot simply go there and tell them that she cannot beat ...*(E 10). Not withstanding the fact that the Unit is part of the territory of the community, the practitioners expressed that the violence *exists but does not get them* (Gp2). The Emergency and fast-track care Units give frequently care to violent situations against children and adolescents and usually in more serious conditions: The practitioners linked this issue to the fact of the distance from the territory and " impersonality" 

The house visits is included in PHC as a technology of care; it is an important tool for the health teams for been integrated and having knowledge of the actual context of the population's living conditions, promoting the linkage and comprehension of family dynamics^16^ ^-^ ^17^. A study stresses the fact that emancipatory practices as the house visits, allow for apprehending the health conditions as a result of the social relations, empowering other therapeutic devices such as team work and community participation^18^. In spite of being a kind of care that all the practitioners may perform, the house visits are mainly done by CHW's: *The investigation is only of the CHWs... I think that if I win I would look like the CSI... (Gp1)*. Other aspect that the study proved about the CHW was a certain isolation of them in the healthcare, leading to propose a integration of them to the teams to overcome this trend^19^. 

On the last quoted speech, the house visits in the network of care for IVCA have the role of "investigation", control and surveillance in a police fashion. Dialogically, the topic presents itself as key in the approach to IVCA, allowing to find situations that would hardly been evidenced in Unit visits, along with the perception of violent family dynamics. This approach is difficult and threatening, specially for the CHW: *There is a lot of fear of accusation, and even when we have a charge, there is fear of dealing with something deeply hidden, there is a lot of fear, even the CHW's are frightened, I live here and I may be threatened* (E4). 

In the family context, power is directed towards the household responsible person and the entry or intervention of a health practitioner, or of who is dialogically his/her neighbor, may provoke the surfacing of a asymmetric relation. This question relates to the issue of multiplicity and plurality of territories where the Unit has to act upon. It is understood as multiplicity the relations imbricated in this care, while plurality may be understood as the set of contexts that need to be incorporated as inherent for this care^14^ ^-^ ^15^. The health practitioners need to approach to this debate and develop their practices around these concepts, building a multidimensional point of view. A study in a Brazilian city stresses the action of PHC practitioners in populations with violent situations as with narrow and not integrative points of view^3^. The literature also stresses the need of a paradigm shift for coping with violence against children and adolescents, suggesting the evaluation of the family dynamics of closeness or violence from a relational view, in this context and the relations of this with the other contexts in which the family participates^20^. 

In the sites of this study, drug traffic appears to be a mobilizing force in the community, and especially in relation to IVCA, posing as "carers" of the victims and the legitimate and symbolic power in the territory of the Unit: *Unfortunately the one that protected, that stopped violence at that point, in that family with the children is the drug traffic...not the police...(...) He came and said - if you raise your hand again on that boy, you're dead!* (Gp5). The literature is in its early stages in the approach to this topic, showing that the discussion on drug trafficking and its links with the community still needs to dig deeper. It is understood that the traffic emerges, as per the speeches of the group, in the inefficiency or insufficiency of the State to respond to the population needs. In many communities, drugs, weapons and violence constitute a large portion of the economic and political infrastructure^21^. It is an important appropriation and domination of the territories, resulting in power relationships that are legitimated by the community, even being informal^21^.

Other than these considerations, the interviewees referred the hardships of convivial with this power of drug traffic in the territories of care of the community, unveiling the contradictions of this relation: *I don't have safety, I work with the power relationship, right? We stay here with these hardships and frightened to deal with this relationship, it's not like: easy! Just notify and that's it... you can receive this mother, that child, that teenager is complicated... even because is in this block, the main drug traffic is here, you know?* (Gp 2). The practitioners say that children and teenagers have early involvement with drug traffic usually in family violence situations as a "scape route". The literature remarks that violence exposure in teenagers and specially family and community violence, rises the risk for future use of marihuana and alcohol^23^. A study in the United States warns for the considerable rise in depression in the involvement with gangs and infection by sexually transmitted diseases by teenagers that break the law and live in deprived social milieus (For example a weakened social network, precarious housing, exposure to violent communities, limited schooling opportunities)^24^. 

Interactions with drug traffic in all the circumstances, in which they may happen, can be considered dialogic with dynamic movements of order and disorder, surrounded by antagonisms and contradictions. The recursivity in this phenomenon shows the complexity around it, meaning that there is not a perceived a cause-effect rationale, but are seen simultaneously as products and producers of the violence that produced it at the first time and retroact on it^8^ ^-^ ^9^. Integrative and inter-sectoral programs and actions focused in prevention, as mentioned before, are imperatives for creating "incubators" for growth and knowledge in cities in the industrialized and developing world, going against the present production of cycles of violence and misery^21^. 

The community is seen as a partner and with significant linkages with the HBU mainly by its legitimacy in caring for families involved in IVCA: *By the team, right? We are well connected, right*?... (Gp3*) Because the family seeks it a lot, I think that we are a good reference for the family, we have a trust link right?... For everything right*?...(Gp5). The HBU's see the local Council as a significant representative of the community, for care directed to families involved in IVCA: *Our local council participates in these cases, they bring everything here. In that way we may be aware of the situation* (Gp 3) 

The values that are cultivated in the world nowadays have increased the rationalist individualism, helping to disintegrate the feeling of belonging to a community and inhibiting potential solidarity^8^. The practitioners reports showed the existence of community practices that act as main factors protecting the families involved in IVCA and have direct implications in the care they deliver: *Because you live there in that little world, staying one in other's home, to help... Solidarity... among the humblest*...(Gp5). In a recent literature review, with reference to the ecological model to approach interpersonal violence as proposed by the World Health Organization^1^, the experiences of countries such as Uganda, India and Nicaragua showed the relevance and efficiency of the community-based strategies for facing violence against women and adolescents. Those strategies propose shifts in the mindsets of power and gender, as a stepping-stone to provoke changes in individual and social relations^25^. 

The complex thinking tries to re-link the dimensions individual/species/society that give foundations to the ethics of solidarity. This issue is key for the debate about health promotion in the collective sphere, where is sought a better interaction among individuals of the community, giving rise to healthy experiences and relationships^3^ ^-^ ^9^. These aspects are reinforced by the literature, showing the importance of the empowerment of the community through programs focused in the prevention of harm, and improving the population's participation, and specifically teenagers' in the formulation of specific public policies^5^ ^-^ ^11^.

## Conclusion

According with the present study, it was understood that there was a need of building a way to look to the territory beyond the specific geographic boundaries, to allocate population to services. The study opened a discussion about the multiplicity and plurality of territories that are in focus when designing the system of care for IVCA through PHC. In this environment, the CHW's were the essential elements for discussion; even though they were considered the key point for PHC, the dialogic as experienced by these practitioners remains to be better understood. The proximity and the significant relations with the families involved in IVCA make possible the activities of health promotion and prevention of harmful events, acting to foster the development of autonomy and the strengthening of community links, leading to individual and collective empowerment. 

The main limitation of the present study was related to the difficulty in digging deeper in such a specific and differentiated territory as the municipality under study. There was a need to complementarity of information such as the point of view of the families when analyzing the findings. As a starting point, it is recommended to build new studies that focus on the views of children, teenagers, families and communities about and facing IVCA, seeking for new contributions that may look to the present procedures under a different light and allow the emergence of the multidimensionality of the phenomenon.

## References

[1] Word Health Organization (2014). Global status report on violence prevention.

[2] Ministério da Saúde (2011). Secretaria de Atenção à Saúde. Departamento de Ações Programáticas e
Estratégicas. Metodologias para o cuidado de crianças, adolescentes e famílias em
situação de violências.

[3] Apostólico MR, Nóbrega CR, Guedes RN, Fonseca RMGS, Egry EY (2012). Characteristics of violence against children in a Brazilian Capital
Rev. Latino-Am. Enfermagem.

[4] Fry DA, Messinger AM, Rickert VI, O'Connor MK, Palmetto N, Lessel H (2013). Adolescent relationship violence help-seeking and help-giving
behaviors among peers. J Urban Health.

[5] Mikton C, MacMillan H, Dua T, Betancourt TS (2014). Integration of prevention of violence against children and early child
development. Lancet Glob Health.

[6] Foshee VA, Benefield TS, Reyes HL, Ennett ST, Faris R, Chang LY (2013). The peer context and the development of the perpetration of adolescent
dating violence. J Youth Adolesc.

[7] Karriker-Jaffe KJ, Foshee VA, Ennett ST (2011). Examining how neighborhood disadvantage influences trajectories of
adolescent violence a look at social bonding and psychological
distress. J Sch Health.

[8] Morin E (2005). Ciência com consciência. Tradução de Maria D. Alexandre e Maria Alice Sampaio
Dória. 8 ed.

[9] Morin E (2007). Introdução ao pensamento complexo.

[10] Pádua EMM (2015). Complexidade e Pesquisa Qualitativa aproximações. Série Acadêmica, PUC-Campinas.

[11] Monteiro EM, Neto WB, Lima LS, Aquino JM, Gontijo DT, Pereira BO (2015). Culture Circles in adolescent empowerment for the prevention of
violence. Int J Adolesc Youth.

[12] Oliveira M, Cruz N, Moura L, Moura J, Coelho R, Melo M (2015). Care to pregnant adolescents perspectives and performance of community
health agents. Rev Enferm UERJ.

[13] Lopes DMQ, Beck CLC, Prestes FC, Weiller TH, Colomé JS, Silva GM (2012). Community Health Agents and their experiences of pleasure and distress
at work a qualitative study. Rev Esc Enferm USP.

[14] Haesbaert R (2013). Del mito de la desterritorialización a la
multiterritorialidad. Cultura y representaciones sociales.

[15] Moraes DE, Canôas SS (2013). O Conceito de "Território" e seu significado no campo da Atenção
Primária à Saúde. Rev Desenvol Soc.

[16] Albuquerque ABB, Bosi M (2009). M Visita domiciliar no âmbito da Estratégia Saúde da Família:
percepções de usuários no Município de Fortaleza, Ceará, Brasil. Cad Saúde Pública.

[17] Cunha MS, Sá MC (2013). A visita domiciliar na estratégia de saúde da família os desafios de
se mover no território. Interface. (Botucatu).

[18] Campos CMS, Silva BRB, Forlin DC, Trapé CA, Lopes IO (2014). Emancipatory practices of nurses in primary health care the home visit
as an instrument of health needs assessment. Rev Esc Enferm USP.

[19] Onocko-Campos RT, Campos GWS, Ferrer AL, Corrêa CRS, Madureira PR, Gama CAP (2012). Evaluation of innovative strategies in the organization of Primary
Health Care. Rev Saúde Pública.

[20] Frewen P, Brown M, DePierro J, D'Andrea W, Schore A (2015). Assessing the family dynamics of childhood maltreatment history with
the Childhood Attachment and Relational Trauma Screen (CARTS) Eur. J. Psychotraumatol.

[21] Ward E, Ashley D (2013). The New Imperative Reducing Adolescent-Related Violence by Building
Resilient Adolescents. J Adolesc Health.

[22] Lobato GR, Moraes CL, Nascimento MC (2012). Desafios da atenção à violência doméstica contra crianças e
adolescentes no Programa Saúde da Família em cidade de médio porte do estado do
Rio de Janeiro, Brasil. Cad Saúde Pública.

[23] Wright EM, Fagan AA, Pinchevsky GM (2013). The Effects of Exposure to Violence and Victimization across Life
Domains on Adolescent Substance Use. Child Abuse Negl.

[24] Voisin DR, Sales JM, Hong JS, Jackson JM, Rose ES, DiClemente RJ. (2015). Social Context and Problem Factors among Youth with Juvenile Justice
Involvement Histories. Behav Med.

[25] Michau L, Horn J, Bank A, Dutt M, Zimmerman C (2015). Prevention of violence against women and girls lessons from
practice. Lancet.

